# Mentored training and its association with dissemination and implementation research output: a quasi-experimental evaluation

**DOI:** 10.1186/s13012-020-00994-0

**Published:** 2020-05-11

**Authors:** Rebekah R. Jacob, Angeline Gacad, Margaret Padek, Graham A. Colditz, Karen M. Emmons, Jon F. Kerner, David A. Chambers, Ross C. Brownson

**Affiliations:** 1 Prevention Research Center in St. Louis, Brown School, Washington University in St. Louis, One Brookings Drive, Campus, Box 1196, St. Louis, MO 63130 USA; 2 Department of Surgery, Division of Public Health Sciences, Alvin J. Siteman Cancer Center, Washington University School of Medicine, Washington University in St. Louis, St. Louis, MO USA; 3 Harvard T.H. Chan School of Public Health, Boston, MA USA; 4 Canadian Partnership Against Cancer, Toronto, Ontario Canada; 5 Division of Cancer Control and Population Sciences, National Cancer Institute, Bethesda, MD USA

**Keywords:** Evaluation, Dissemination and implementation, Scientific productivity, Dissemination and implementation research training

## Abstract

**Background:**

There is a continued need to evaluate training programs in dissemination and implementation (D&I) research. Scientific products yielded from trainees are an important and objective measure to understand the capacity growth within the D&I field. This study evaluates our mentored training program in terms of scientific productivity among applicants.

**Methods:**

Post-doctoral and early-career cancer researchers were recruited and applied to the R25 Mentored Training for Dissemination and Implementation Research in Cancer (MT-DIRC) between 2014 and 2017. Using application details and publicly available bibliometric and funding data, we compared selected fellows with unsuccessful applicants (nonfellows). We extracted Scopus citations and US federal grant funding records for all applicants (*N* = 102). Funding and publication abstracts were de-identified and coded for D&I focus and aggregated to the applicant level for analysis. Logistic regression models were explored separately for the odds of (1) a D&I publication and (2) US federal grant funding post year of application among fellows (*N* = 55) and nonfellows (*N* = 47). Additional models were constructed to include independent variables that attenuated the program’s association by 5% or more. Only US-based applicants (*N* = 87) were included in the grant funding analysis.

**Results:**

Fellows and nonfellows were similar across several demographic characteristics. Fellows were more than 3 times more likely than nonfellows to have grant funding after MT-DIRC application year (OR 3.2; 95% CI 1.1–11.0) while controlling for time since application year; the association estimate was 3.1 (95% CI 0.98–11.0) after adjusting for both cancer research area and previous grant funding. For publications, fellows were almost 4 times more likely to publish D&I-focused work adjusting for time (OR 3.8; 95% CI 1.7–9.0). This association lessened after adjusting for previous D&I publication and years since undergraduate degree (OR 2.9; 95% CI 1.2–7.5).

**Conclusions:**

We document the association of a mentored training approach with built-in networks of peers to yield productive D&I researchers. Future evaluation efforts could be expanded to include other forms of longer-term productivity such as policy or practice change as additional objective measures. D&I research trainings in the USA and internationally should consider common evaluation measures.

Contributions to the literature

- The number of trainings in dissemination and implementation (D&I) continues to expand globally. Few D&I training evaluations are published, and those evaluations reported tend to be small-scale and short term with limited measures of impact.
- This study reports that our training program enhances academic productivity and highlights mentored training for D&I scholars as an essential approach for building capacity for D&I research.
- Using publicly available data, the methods in this study could be replicated with other D&I trainings to compare impact across fields.

## Background

Growing the field of dissemination and implementation (D&I) research and advancing the next generation of individuals to lead it are high priorities [1, 2]. These goals have been supported recently by several training programs across the globe which provide researchers with a solid foundation in approaches to close the gap between scientific discovery and practice. The programs vary in breadth and focus (mental health, cancer, etc.) as well as format (in-person, web-based, etc.) and content delivery components (didactic, on-site visits, mentoring, etc.) [3–14].

Much of what we know about the potential benefits of these trainings is from examining how participants fared after the training either by comparing pre-post knowledge or competency needed to grow the D&I field. However, few D&I training programs have examined the trajectory of the training participant with respect to peers who did not receive the same training. Typically, training programs are not randomized by design, and understanding effects is challenging. Recently, two D&I training programs introduced unsuccessful applicants as a comparison group to evaluate research productivity resulting from program participation. The evaluation of the Training Institute for Dissemination and Implementation Research in Health (TIDIRH) combined cross-sectional surveys of past fellows with portfolio analyses of grants submitted and funded [15]. In addition, the mental health-focused Implementation Research Institute (IRI) program studied bibliometric outcomes among fellows and non-selected applicants [16]. Other academic training efforts have also begun to take advantage of the growing availability of publicly accessible research product information [17, 18]. Such studies pave the way for a meaningful understanding of the associations between mentored training and scientific productivity. The previous studies also provide valuable insights to funders, especially within the context of learning how best to train and build capacity for a newer field such as D&I science.

The Mentored Training for Dissemination and Implementation Research in Cancer (MT-DIRC) has trained and mentored over 50 junior to mid-level career researchers to increase their knowledge of and capacity for D&I research in cancer. The training increased D&I research competencies from pre- to 6- and 18-months post their initial training [6]. The purpose of this study is to examine the program’s association with capacity building for D&I research, specifically in the areas of publishing and acquiring US Federal Grant funding for D&I research.

## Methods

This study used a quasi-experimental design (pre/post with a comparison group) to examine the relationship of the MT-DIRC training program with subsequent scholarly output in the form of D&I-related publications and US federal funding secured for research. Our main research question sought to determine if there was a difference among applicants in the likelihood to produce research outputs after participation (vs. nonparticipation) in the program.

### Training program description summary

The MT-DIRC program was an R25 mentored training funded by the National Cancer Institue (PAR-12-049) to increase the capacity for D&I research related to the spectrum of cancer control (etiology to survivorship) [19]. The full description of the program is published with preliminary results on skill building [6]. Here, we provide a brief summary of the program.

Eligibility requirements included a doctorate degree and full-time appointment in a research setting. Recruitment through various listservs and advertisements at D&I-related events and conferences focused mostly on early-career researchers or mid-to-late-career researchers looking to shift the focus of their work to D&I research. To apply, researchers submitted an informational cover page, concept paper for a D&I study to work on during the program, biosketch, and two letters of reference. Program faculty rated each application based on several areas including the overall application quality, commitment to D&I research, experience working in trans-disciplinary networks, research support, likelihood for career development, appropriate methods in the concept paper, appropriate topic in the concept paper, and potential impact of the work proposed.

Selected applicants participated in two Summer Institutes (5-day trainings) in St. Louis, MO, USA, each June. Trainings primarily focused on competencies for D&I research [20] and in-person mentoring and interactive sessions to work on and receive feedback related to research proposals and or other projects in progress. Ongoing evidence-informed mentoring [21], often in the form of regularly scheduled calls, continued for 2 years. Fostering collaboration among the program’s network of fellows and mentors was a concerted focus of the program. All current and past fellows and mentors were invited to participate in quarterly webinars and to attend annual meet-up events at the D&I science conference in Washington, DC.

### Data collection and processing

In total, 105 applicants applied to the program between 2014 and 2017. Three who were selected into the program later dropped out for various career/personal reasons and were excluded from this study. The total sample of program applicants included in this study is 102: 55 that were selected and participated in the MT-DIRC program (“fellows”) and 47 unselected applicants (“nonfellows”) as the comparison group. Demographic information data was collected from each application.

For information on scholarly output, two main sources of publicly available data were used. To gather all published works, we utilized Scopus (www.scopus.com), a comprehensive citation database with over 75 million records [22]. All applicants to the MT-DIRC program were searched in the Scopus database, and after verifying academic affiliation, their citations and accompanying abstracts were extracted through Scopus’s BibTeX export tool in July 2019 and further processed in R using the “bibliometrix” package (2.3.2) [23]. A total of 5189 publication citations were extracted. Initially, 11% (*N* = 565) were missing abstracts. Upon closer examination, certain article types were responsible for much of the missing abstracts and were necessary to exclude (erratums = 30, notes = 208, letters = 129). For the remaining with missing abstracts (*N* = 258), a member of the research team searched each citation to confirm missing abstracts (e.g., some journals do not require) or extract the abstract if found. A total of 208 additional abstracts were located, and the remaining citations without abstracts (*N* = 50) were excluded. The final dataset contained 4772 citations and included only citation ID number, applicant ID number, title, and abstract for further de-identified coding.

To gather grant funding, we used the National Institutes of Health’s (NIH) Research Portfolio Online Reporting Tools (RePORTER Tool Manual, 2018). RePORTER is an electronic tool to search the US federal repository of intramural and extramural NIH-funded research projects dating back to 1985. The repository also includes funded projects by the Administration for Children and Families (ACF), the Agency for Healthcare Research and Quality (AHRQ), the Centers for Disease Control (CDC), the Health Resources and Services Administration (HRSA), the Food and Drug Administration (FDA), and the Department of Veteran Affairs (VA). The R package “fedreporter” (0.2.1) [24] was used to extract grant funding information from RePORTER’s application programming interface (API) including abstracts for all applicants in September 2019. We included records where the applicant was either a coinvestigator or a principal investigator. A total of *N* = 271 funding records were extracted. After keeping the first fiscal year of duplicate entries (e.g., multi-year funding), the total sample of unique US federally funded projects was *N* = 97. Funding cases were reduced to grant ID, applicant ID, title, and abstract for further de-identified coding.

#### Coding process

For each publication and grant abstract, D&I focus was coded as “yes” or “no” similar to Baumann and colleagues’ criterion [16]. D&I focus “yes” included an “implementation-centered hypothesis, design, or framework, focused on assessing the implementation climate of an organization, or described the implementation processes for a particular intervention.” For example, abstracts which explicitly examined the implementation outcomes were coded as “yes,” and those that focused only on the intervention outcomes were coded as “no.” In addition, all publications featured in the *Implementation Science* journal were coded as D&I “yes” based upon the journal’s inherent D&I focus and scope. Two project staff (RRJ, AG) coded the publication and grant abstracts. For publications, an initial random selection of citations (*N* = 20) was double coded by both project staff and discussed to reach agreement on the inclusion and exclusion criteria before proceeding with single coding by one coder (AG) for the remaining citations. A random sample of 10% was selected for double coding and showed a 93% agreement. The number of grants was considerably less than the total number of publications, and therefore, all were double coded with any discrepancies reconciled to reach 100% consensus.

## Data analysis

After coding was completed on each publication and grant, data were aggregated to the applicant level and merged with the applicant demographic data for analysis (*N* = 102). Because RePORTER is solely for US-based research, we excluded foreign applicants (*N* = 15) from the grant analyses.

We compared demographic, publication, and funding data by applicant status with independent samples *t* tests (continuous data) and chi-square tests (categorical data). For modeling, two main binary outcomes were examined: any D&I publication after the application year (yes or no), and because the program supported grant writing in general, “any” grant funding after the application year (yes or no). Binary logistic regression models examined program participation status with each outcome. Attenuation or change in estimates (CIE) of program participation resulting from the inclusion of independent variables was examined [25, 26]. Variables which resulted in more than 5% attenuation were included in separate and combined models for further examination. All analyses were completed in R [27] with an alpha level set at 0.05.

## Results

Fellows and nonfellows were similar across several demographic characteristics (Table 1). The majority were female (80.4%), and “prevention” was the most common cancer research area focus (48.0%). Assistant professor was the largest category of position among applicants (46.1%) followed by post-doctoral or research scientist (21.6%), others (17.6%), and associate professor or professor (14.7%). On average, applicants were 17.9 (SD = 6.7) years post-undergraduate degree when they applied for the MT-DIRC program.

**Table 1:** Applicant characteristics (*N* = 102), Mentored Training for Dissemination and Implementation Research in Cancer

	Total (*N* = 102), *N* (%)	Fellow (*N* = 55), *N* (%)	Nonfellow (*N* = 47), *N* (%)	*p* value^a^
Gender				0.914
Female	82 (80.4)	44 (80.0)	38 (80.9)	
Male	20 (19.6)	11 (20.0)	9 (19.1)	
Cancer area focus				0.457
Detection	16 (15.7)	11 (20.0)	5 (10.6)	
Prevention	49 (48.0)	24 (43.6)	25 (53.2)	
Survivorship	18 (17.6)	11 (20.0)	7 (14.9)	
Treatment	19 (18.6)	9 (16.4)	10 (21.3)	
Position				0.475
Associate professor or professor	15 (14.7)	10 (18.2)	5 (10.6)	
Assistant professor	47 (46.1)	27 (49.1)	20 (42.6)	
Post-doctoral or research fellow	22 (21.6)	10 (18.2)	12 (25.5)	
Others	18 (17.6)	8 (14.5)	10 (21.3)	
Home institution within the USA				0.242
No	15 (14.7)	6 (10.9)	9 (19.1)	
Yes	87 (85.3)	49 (89.1)	38 (80.9)	
Years from an undergraduate degree at the time of application, mean (SD)	17.9 (6.7)	18.2 (6.3)	17.4 (7.2)	0.565
Application year (cohort)				0.011
2014	21 (20.6)	13 (23.6)	8 (17.0)	
2015	22 (21.6)	15 (27.3)	7 (14.9)	
2016	18 (17.6)	13 (23.6)	5 (10.6)	
2017	41 (40.2)	14 (25.5)	27 (57.4)	

^a^*p* values from independent samples *t* tests for continuous data and Pearson chi-square tests for categorical data

Fellows and nonfellows differed in grant funding before their application year (51.0% vs. 34.2%), and fellows were more likely to have grant funding following the application year (36.7% vs. 13.2%) (Table 2). Before and after the application year, fellows were more likely to have a D&I publication than nonfellows (36.4% vs. 14.9%; 65.5% vs. 31.9%). These differences were accounted for in the multivariate analyses that follow.

**Table 2:** Applicant funding and publication (*N* = 102), Mentored Training for Dissemination and Implementation Research in Cancer

	Total	Fellow	Nonfellow	*p* value^a^
Grant funding in RePORTER (US applicants only, *N* = 87)	*N* = 87	*N* = 49	*N* = 38	
Before or at the application year				
Grant funding, *N* (%)				0.117
No	49 (56.3)	24 (49.0)	25 (65.8)	
Yes	38 (43.7)	25 (51.0)	13 (34.2)	
D&I grant funding, *N* (%)				0.009
No	79 (90.8)	41 (83.7)	38 (100.0)	
Yes	8 (9.2)	8 (16.3)	0 (0.0)	
After^b^ the application year				
Grant funding, *N* (%)				0.013
No	64 (73.6)	31 (63.3)	33 (86.8)	
Yes	23 (26.4)	18 (36.7)	5 (13.2)	
D&I grant funding, *N* (%)				0.042
No	75 (86.2)	39 (79.6)	36 (94.7)	
Yes	12 (13.8)	10 (20.4)	2 (5.3)	
Publications indexed in SCOPUS (all applicants, *N* = 102)	*N* = 102	*N* = 55	*N* = 47	
Before or at the application year				
D&I publication				0.014
No	75 (73.5)	35 (63.6)	40 (85.1)	
Yes	27 (26.5)	20 (36.4)	7 (14.9)	
After^c^ the application year				
Published D&I after^c^ application				<0.001
No	51 (50.0)	19 (34.5)	32 (68.1)	
Yes	51 (50.0)	36 (65.5)	15 (31.9)	

^a^*p* values from Pearson chi-square tests

^b^After represents the time between the application year and September 2019 when data was collected

^c^After represents the time between the application year and July 2019 when data was collected

Table 3 shows that fellows were more than three times more likely than nonfellows to have grant funding after the MT-DIRC application year (OR 3.2; 95% CI 1.1–11.0, model 1) while controlling for time since the application year. Effect estimates were 3.3 (95% CI 1.1–11.6, model 2), adjusting for cancer research area, 3.0 (95% CI 0.99–10.2, model 3) adjusting for previous grant funding, and 3.1 (95% CI 0.98–11.0, model 4) after adjusting for both cancer area and previous grant funding.

**Table 3:** US applicant awarded federally funded grant between the application year and September 2019 (*N* = 87)

	Model 1		Model 2		Model 3		Model 4	
	*b* (SE)	OR (95% CI)	*b* (SE)	OR (95% CI)	*b* (SE)	OR (95% CI)	*b* (SE)	OR (95% CI)
MT-DIRC								
Nonfellow (ref)								
Fellow	**1.2 (0.6)**	**3.2 (1.1–11.0)**	**1.2 (0.6)**	**3.3 (1.1–11.6)**	1.1 (0.6)	3.0 (0.99–10.2)	1.1 (0.6)	3.1 (0.98–11.0)
Time^a^	0.3 (0.2)	1.4 (0.9–2.1)	0.4 (0.2)	1.5 (0.9–2.4)	0.3 (0.2)	1.4 (0.9–2.1)	0.40 (0.2)	1.5 (0.96–2.4)
Cancer research area								
Treatment (ref)								
Survivorship			− 0.9 (0.86)	0.4 (0.1–2.0)			− 0.8 (0.9)	0.5 (0.1–2.6)
Detection			− 0.8 (0.85)	0.5 (0.1–2.4)			− 0.7 (0.9)	0.5 (0.1–2.6)
Prevention			− 1.1 (0.71)	0.3 (0.1–1.3)			− 1.1 (0.7)	0.3 (0.1–1.3)
Previous grant funding								
No (ref)								
Yes					0.6 (0.5)	1.9 (0.7–5.3)	0.6 (0.5)	1.9 (0.7–5.7)

^a^Time in years from the application year to September 2019, when data was collected. Bold values are significant at *p* < 0.05

Table 4 shows that fellows were almost four times more likely to publish D&I-focused work compared to nonfellows while adjusting for time (OR 3.8, 95% CI 1.7–9.0, model 1). Odds remained elevated for fellows after additionally adjusting for previous D&I publication (OR 3.1, 95% CI 1.3–7.7, model 2), years since undergraduate degree (OR 3.5, 95% CI 1.5–8.8, model 3), and both D&I publication and years since undergraduate degree (OR 2.9, 95% CI 1.2–7.5, model 4).

**Table 4:** Applicant published D&I article between the application year and July 2019 (*N* = 102)

	Model 1		Model 2		Model 3		Model 4	
	*b* (SE)	OR (95% CI)	*b* (SE)	OR (95% CI)	*b* (SE)	OR (95% CI)	*b* (SE)	OR (95% CI)
MT-DIRC								
Nonfellow (ref)								
Fellow	**1.3 (0.4)**	**3.8 (1.7–9.0)**	**1.1 (0.4)**	**3.1 (1.3–7.7)**	**1.3 (0.5)**	**3.5 (1.5–8.8)**	**1.1 (0.5)**	**2.9 (1.2–7.5)**
Time^a^	0.1 (0.2)	1.1 (0.8–1.6)	0.1 (0.2)	1.1 (0.8–1.6)	0.1 (0.2)	1.1 (0.7–1.6)	0.1 (0.2)	1.1 (0.7–1.6)
Previous D&I publication								
No (ref)								
Yes			**1.4 (0.5)**	**4.2 (1.5–13.1)**			**1.4 (0.6)**	**4.2 (1.5–1.4)**
Years since undergraduate degree					**− 0.1 (0.0)**	**0.93 (0.86–0.99)**	**− 0.1 (0.0)**	**0.93 (0.86–0.99)**

^a^Time in years from application to July 2019, when data was collected. Bold terms are significant at *p* < 0.05

## Discussion

Overall, our results show that, in general, fellows were more likely than nonfellows to contribute to the D&I research literature following their participation in the MT-DIRC program.

Our findings are similar to the evaluation study of a mentored implementation science training in mental health where fellows were more likely to receive D&I grants and publish D&I work post-fellowship [16]. Comparatively, selected fellows to MT-DIRC were less experienced in D&I with fewer D&I publications (36% vs. 67%) and D&I funding (16% vs. 36%) at baseline than IRI fellows. Unlike the IRI program, MT-DIRC did not require experience writing NIH grants as a prerequisite, which might account for this difference. Nonetheless, both studies point to the career impact and potential for D&I research capacity growth within the field through multi-year, mentored training approaches.

Globally, several training programs aim to build capacity for translational research (translating research to practice and communities). A recent synthesis of US and international D&I research trainings describe a large variety in approaches, ranging from single, short courses to long-term academies that combine didactic training with mentoring [8]. Training endeavors have contributed to the field with evaluation findings, though common metrics are lacking and could aid in understanding the trainings’ collective impact on capacity building. In addition, understanding training outputs (outcomes) based upon training inputs (e.g., frequency, length, format) could identify “active ingredients” for the largest impact. Here, we provide a fairly simple template with publicly available data to understand the training impact on scientific productivity in the field. Attention from the field is needed to develop templates for other impacts or common measures (knowledge, networking, mentoring) from D&I research training programs. Our program also conducted qualitative interviews with fellows to understand how specific activities within the program may have affected these measures (analyses in process). Combined evaluation approaches are likely needed to fully understand the impacts of specific program activities on research outputs.

One byproduct of mentored training approaches is the network of peers and mentors which has been linked to increased research collaborations [28]. Building and fostering collaborations within this network was the main objective of the MT-DIRC-mentored approach to training and may have attributed to the increased scientific productivity of our fellows. Guise and colleagues’ qualitative evaluation with mentors and mentees suggests team mentoring “works when” mentors and mentees bring varying perspectives and expertise and when mentors promote networking among mentees [29]. At the 5-day in-person Summer Institute, fellows at various intersections of D&I cancer prevention and control research connected and organized manuscripts to address gaps in the science. These collaborative publications were also supported and encouraged through in-person mentoring and group mentoring calls. Group mentoring provided a platform to interact with like-minded peers, a dedicated space for critique by experts in the field, and a continuous longer-term relationship with peers and mentors that likely aided fellows in accelerating their research progress.

Generating manuscripts is often accomplished in a shorter time frame in comparison with the process of proposing and receiving competitive grant funding. At the time of this study, it has been 5 years since the first cohort attended their first Summer Institute. Producing and disseminating key research in D&I is an important step to increase knowledge and fill gaps of understanding. This speaks to the potential of a mentored training program to accelerate D&I capacity building at a relatively rapid pace, which is needed to continue to build the field.

## Limitations

While our study provides one approach to understanding the relationship between mentored training and building capacity in D&I research, several limitations should be noted. The main limitation is the use of unsuccessful applicants as a control group. While we provide data to show the groups were statistically similar across several characteristics at baseline, there may be unmeasured differences that led fellows to be selected at the outset. An example of this could be applicant selection criteria of research support and potential and likelihood for career development. We did not account for research funding trends which may have given priority for some applicants and not others. We also did not include journal impact measures for citations, a potential factor for understanding research impact. Our 6-year funding period is a function of a grant cycle, and too short for understanding longer-term effects on practice and policy. Easy access to comprehensive productivity information is limited mostly to publications and databases of federal funding. Understanding other forms of productivity like changes to cancer control policy or practice based on D&I research and spreading D&I research capacity through teaching and presenting would be ideal for a fuller understanding of the impact. This would require standardized metrics and measures.

## Conclusions

Mentored training is an important and effective approach to building capacity in D&I research. A growing body of literature shows the value of systematic approaches to mentored training in biomedical research [21] and specifically in D&I research [16]. Our evaluation and other recent literature highlight the combined impacts of didactic teaching, networking, and mentoring. This study used one evaluation approach to examine scientific productivity, and additional steps are needed (e.g., stronger evaluation designs, standardized metrics and measures) to fully document training program impacts.

## Data Availability

The datasets analyzed during the current study are available from the corresponding author upon reasonable request.

## Abbreviations

D&I: Dissemination and implementation
MT-DIRC: Mentored Training for Dissemination and Implementation Research in Cancer
